# Accelerating Lipid Flip-Flop at Low Concentrations: A General Mechanism for Membrane Binding Peptides

**DOI:** 10.1021/acs.jpclett.3c01284

**Published:** 2023-07-31

**Authors:** Manuel Carrer, Josefine Eilsø Nielsen, Henrique Musseli Cezar, Reidar Lund, Michele Cascella, Thereza A. Soares

**Affiliations:** † Department of Chemistry, University of Oslo, Postboks 1033 Blindern, 0315 Oslo, Norway; ‡ Department of Chemistry, University of São Paulo, 055508−090 Ribeirão Preto, Brazil; § Hylleraas Centre for Quantum Molecular Sciences, University of Oslo, 0315 Oslo, Norway

## Abstract

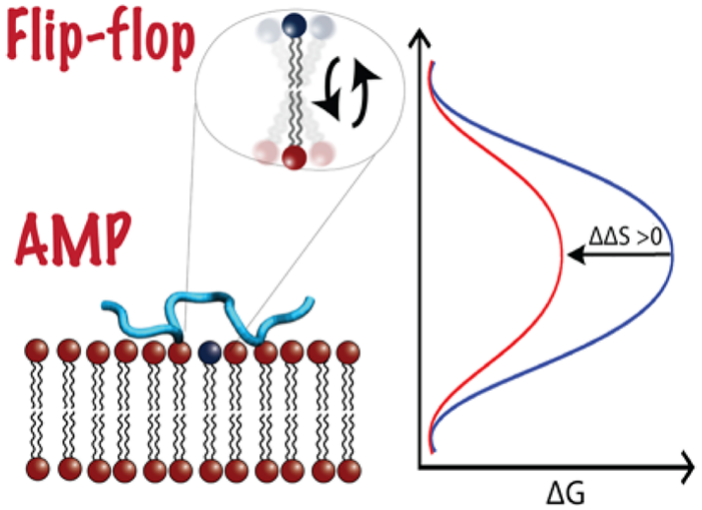

We report a physicochemical investigation of the lipid transport properties of model lipid membranes in the presence of the antimicrobial peptide indolicidin through comparisons of experimental SANS/SAXS scattering techniques to fully atomistic molecular dynamics simulations. In agreement with the experiment, we show that upon peripheral binding of the peptides, even at low concentrations, lipid flip-flop dynamics is greatly accelerated. Computer modeling elucidates the interplay between structural changes and lipid dynamics induced by peptides and proposes a mechanism for the mode of action of antimicrobial peptides, assessing the major role of entropy for the catalysis of the flipping events. The mechanism introduced here is universal for all peptides with preferential peripheral binding to the membrane as it does not depend on the specific amino acid sequence.

Cellular plasma membranes not only define the boundary of cells but also harbor a variety of processes essential for cell homeostasis, intercellular signaling, and molecular exchanges with the environment. Biological membranes are not static entities but exhibit a wide range of dynamic behaviors, from large scale shape fluctuations to local diffusion of individual molecules. Lipid molecules readily exchange laterally within a membrane leaflet (∼0.75 nm, ∼10^7^ times per second), which means that, on average, they diffuse the length of a large bacterial cell (∼2 μm) in about 1 s.^1^ In contrast, lipid molecules seldom undergo flip-flop displacements between leaflets in the bilayer due to the large enthalpic barrier associated with the translocation of lipid hydrophilic heads across the hydrophobic membrane core. Hence, lipid exchange across the membrane leaflets generally involves the activity of flippases (inward moving), floppases (outward moving), and scramblases (bidirectional) enzymes.^2^

Flip-flop events are also facilitated by local membrane defects,^3^ which can be introduced by binding of certain membrane-active compounds, as for instance antimicrobial peptides (AMPs).^4−10^ While most antibiotics target the bacterial cell by blocking specific biochemical pathways, AMPs are reported to have a less specialized mode of action. The precise microscopic mechanism of action of AMPs is a subject to debate. However, their interaction with the lipid membrane is a key feature.^11^ The generally accepted hypothesis involves the formation of AMP induced pores in the cytoplasmic membrane,^12^ leading to leakage of fluids, ions, and other essential molecules through the membrane and eventually to cell death. However, as pointed out by, among others, Wimley, the nature of these pores remains unclear.^13^ For instance, AMPs may create *transient* pores, thus perturbing the membrane and accelerating the transport across the bilayer without the necessity of defined channels.

Recently, using time-resolved small-angle neutron scattering (TR-SANS), some of us showed that a series of naturally derived AMPs significantly accelerate lipid dynamics despite exhibiting very different mode of insertion in the membrane.^14^ Using small-angle X-ray scattering (SAXS), it was observed that peptides such as indolicidin and cecropin A only insert at the membrane surface, while other peptides such as LL-37 and aurein 1.2 are able to penetrate the bilayer. Nevertheless, all of the membrane interacting peptides exhibited similar acceleration of both flip-flop and exchange (intervesicular) dynamics. The enhanced lipid flip-flop process may have a detrimental effect on the cell as it can lead to scrambling of the natural asymmetric lipid composition between the inner and outer membrane leaflet, and couple with ion transport. Perhaps surprisingly, upon analysis of the Arrhenius-like temperature dependence of the flip-flop process, it became evident that the acceleration can be mainly attributed to entropic effects rather than a modification of the enthalpic barriers.

Indolicidin is a small cationic AMP formed by 13 amino acid residues and is disordered in solution. When in contact with membranes, it binds without adopting an α-helical conformation like most natural AMPs.^9,15^ Therefore, indolicidin does not fit into the classical pore model of helices that bundle together and insert perpendicularly into the membrane to form either a barrel-stave or toroidal pore.^15^ In previous studies, using SAXS/SANS together with neutron reflectometry, we found that indolicidin rather inserts itself in the interface between the head and tail group of the outer leaflet^16,17^ supporting the *interfacial activity* model,^13^ where the insertion of the peptide into the polar portion of the bilayer alters the packing of the tails and leads to the disruption of the permeability barrier imposed by the hydrocarbon core in the membrane.

In this Letter, we provide a detailed physicochemical investigation of the lipid transport properties of model lipid membranes in the presence of indolicidin through comparisons of experimental SANS/SAXS scattering techniques with fully atomistic molecular dynamics (MD) simulations. We elucidate the interplay between structural changes and lipid dynamics induced by peptides and propose a mechanism for the mode of action of AMPs by assessing the role of the enthalpic and entropic contributions due to the interaction. We show that upon peripheral binding of the peptides, even at low concentrations, lipid flip-flop dynamics is accelerated. We previously established that peptide-induced acceleration is inherent for a wide range of natural AMPs^9^ and also takes place in *Escherichia coli* mimicking phosphatidylethanolamine lipid mixtures.^10,14,17^ We therefore argue that the mechanism is not only a general feature of AMPs that have the cytoplasmic membrane as primary target, but also of other peptides that interact with the bilayer.

## Peptide Insertion and Membrane Structure

**Figure 1:**
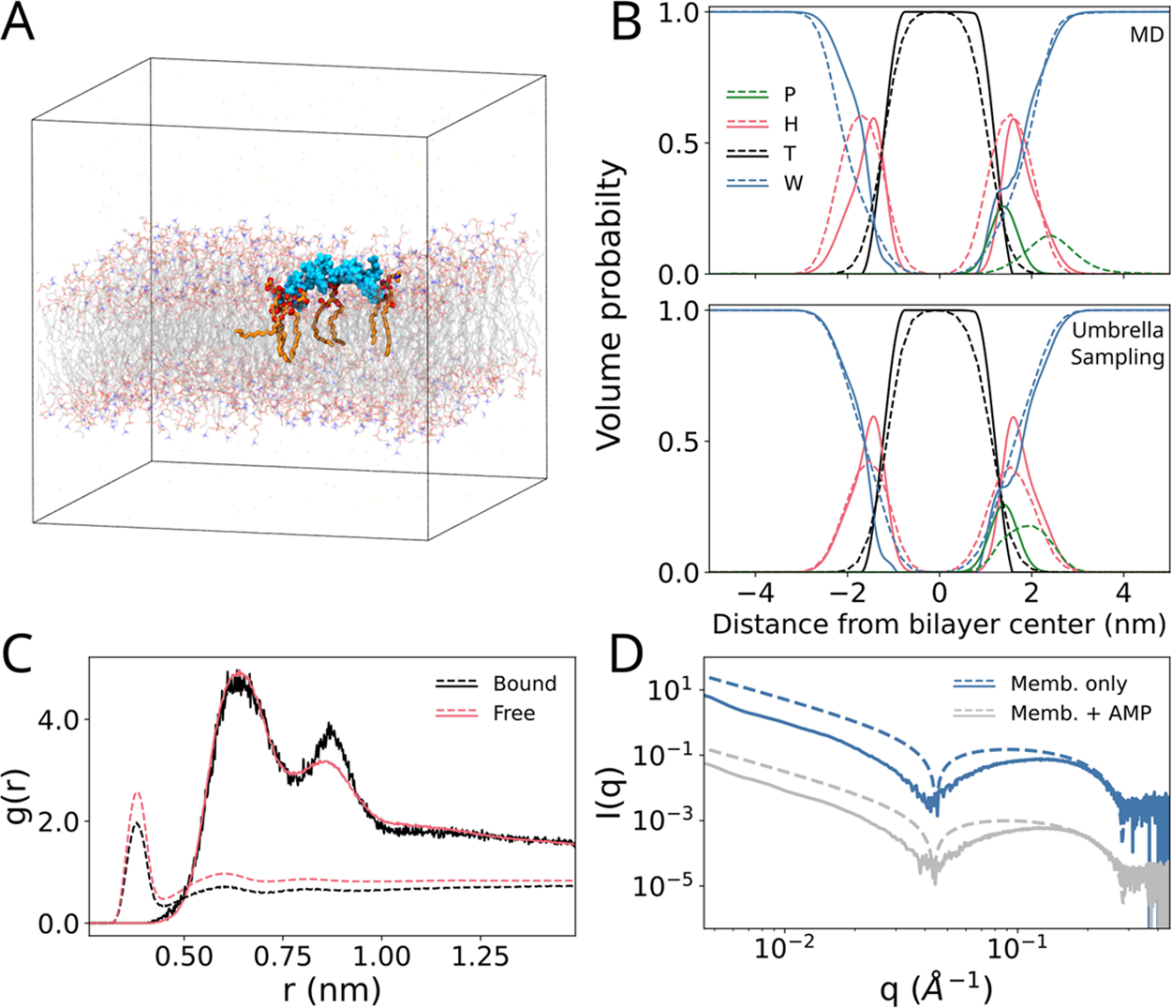
Structure of indolicidin bound to the lipid bilayer. The simulation box showcased in A has one peptide represented in cyan spheres. The lipids forming salt bridges with the peptide are highlighted in orange. The rest of the membrane is rendered in lines. Water is not shown for clarity. (B) Comparison of density profiles obtained from model fits of SAXS data^17^ (solid) and simulations (dashed). These are obtained from unbiased MD (top), and the umbrella window corresponding to the deepest peptide insertion without free energy costs (bottom). The functional groups are represented in green (peptide), red (lipid heads), black (lipid tails), and blue (water). The peptide lines are rescaled by 5× for the experiment and 10× for the simulation for better visualization. The error of the SAXS fits that form the basis of the density profile was found to be less than 5%.^17^ (C) Radial distribution function plots for phosphate–phosphate (solid) and phosphate–water (dashed) distances, from MD simulations. The black line corresponds to the lipid phosphates interacting with the AMP, while the red line is computed for those not interacting with the peptide. (D) Comparison between experimental^17^ (solid) and simulated (dashed) SAXS spectra of the pure membrane and of the membrane with indolicidin.

We performed classical MD simulations of a system composed of a single indolicidin peptide bound to a model 3:1 1,2-dimyristoyl-sn-glycero-3-phosphocholine (DMPC)/1,2-dimyristoyl-sn-glycero-3-phospho-(1′-rac-glycerol) (sodium salt; DMPG) bilayer (Figure 1A) at 303.15 K, a temperature well above the crystalline gel transition for this system, as verified by differential scanning calorimetry (DSC).^9,17^ This composition is in close agreement with the experiment, lacking only the fraction of pegylated lipids used to avoid the formation of multilamellar structures. Initially, being amphipathic, indolicidin floats at the membrane–water interface, anchored to the polar region of the bilayer. We report the normalized density profiles obtained from 1 μs standard MD simulation compared with those extrapolated from SAXS measurements (see ref (17) for details on experimental setup and fit analysis). Computed density profiles converge within 1% after ∼600 ns of simulation time. The SAXS profiles computed from the simulations using the SIMtoEXP software^18^ are in fair agreement with the experimental ones, indicating a reliable model for the lipid packing (Figure 1D). Indolidicin is more hydrated in the molecular model than in the experiment, residing slightly farther from the hydrophobic region of the membrane. The MD peptide volume probability is centered at ∼2.3 nm, compared to ∼1.6 nm in the experiment. This discrepancy may be caused by both too small sampling times and the spurious stronger surface tension caused by periodic boundary conditions. A previous study on the binding of indolicidin to a bilayer with the same lipid composition estimated the optimal binding distance at roughly 2.5 nm from the center of membrane,^19^ while another one instead reported the minimum to be located at around 1.4 nm on a pure POPG bilayer,^20^ which fits better SAXS data, even though it was obtained for a membrane with different lipid composition.

**Figure 2:**
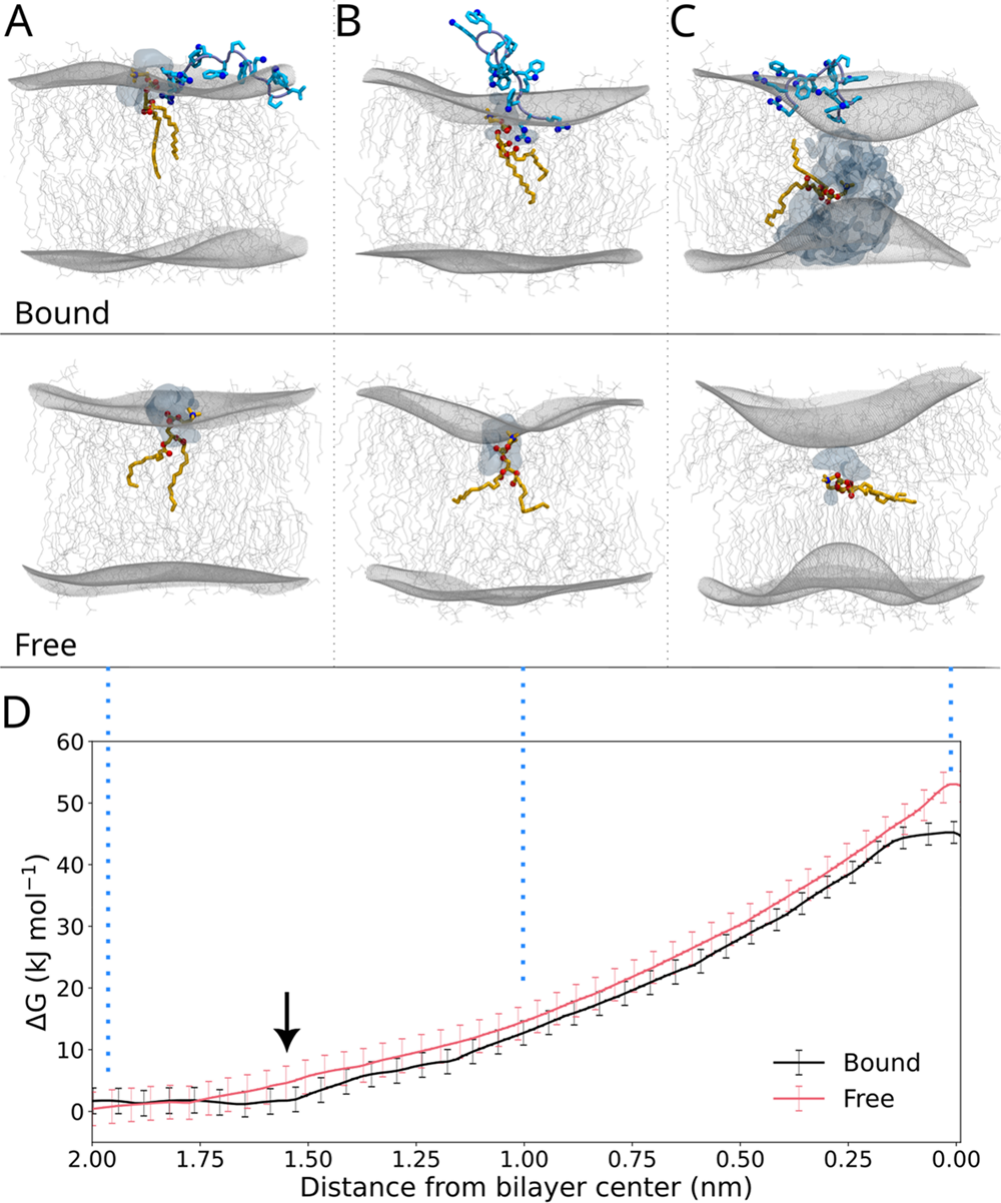
In A, B, and C, selected conformations of the simulated system depict the lipid flipping across the two leaflets: membrane containing indolicidin (top) or in the absence of the peptide (bottom). Isosurfaces delineate water penetrating the bilayer with the flipping lipid. Wired surfaces depict the bilayer leaflets. D reports the PMF profiles obtained from umbrella sampling for the flip-flop process in the presence (black) and absence (red) of indolicidin. The arrow indicates the umbrella window used for the density profile in Figure 1B (bottom).

The interaction between the lipids and the peptide locally deforms the lipid packing. Even though the area per lipid is not significantly affected (APL ≈ 0.580(1) nm^2^), the radial distribution functions between phosphate groups with themselves and with water (Figure 1C) signal a lipid crowding effect induced by the peptide, with an increase in the number of second-neighbor head groups accompanied by reduced direct contact with water, a consequence of solvent displacement due to the presence of indolicidin. The flip-flop free energy was reconstructed by umbrella sampling, biasing the position of a flipping DMPC phosphate head interacting with the peptide along the direction normal to the membrane. The resulting potential of mean force (PMF) was compared with the one obtained for flipping a lipid not bound to the peptide (Figure 2D). During the umbrella sampling simulations, we observed that the penetration of the flipping lipid bound to the peptide is barrierless down to a quota ∼1.6 nm from the center of the membrane (Figure 2D, arrow). The ability of indolicidin to partially penetrate the membrane without free energy costs better corroborates the reported experimental distribution centered at quotas lower than the one observed in the unbiased simulation (Figure 1B, bottom).

When bound to the peptide, the activation free energy for the lipid flipping is roughly 16% lower (∼53 kJ mol^–1^ compared to ∼44 kJ mol^–1^), in qualitative agreement with the experimental findings where a reduction of about 13% was found although the absolute values are larger (74–65 kJ mol^–1^ without and with peptide, respectively).^9^

**Figure 3:**
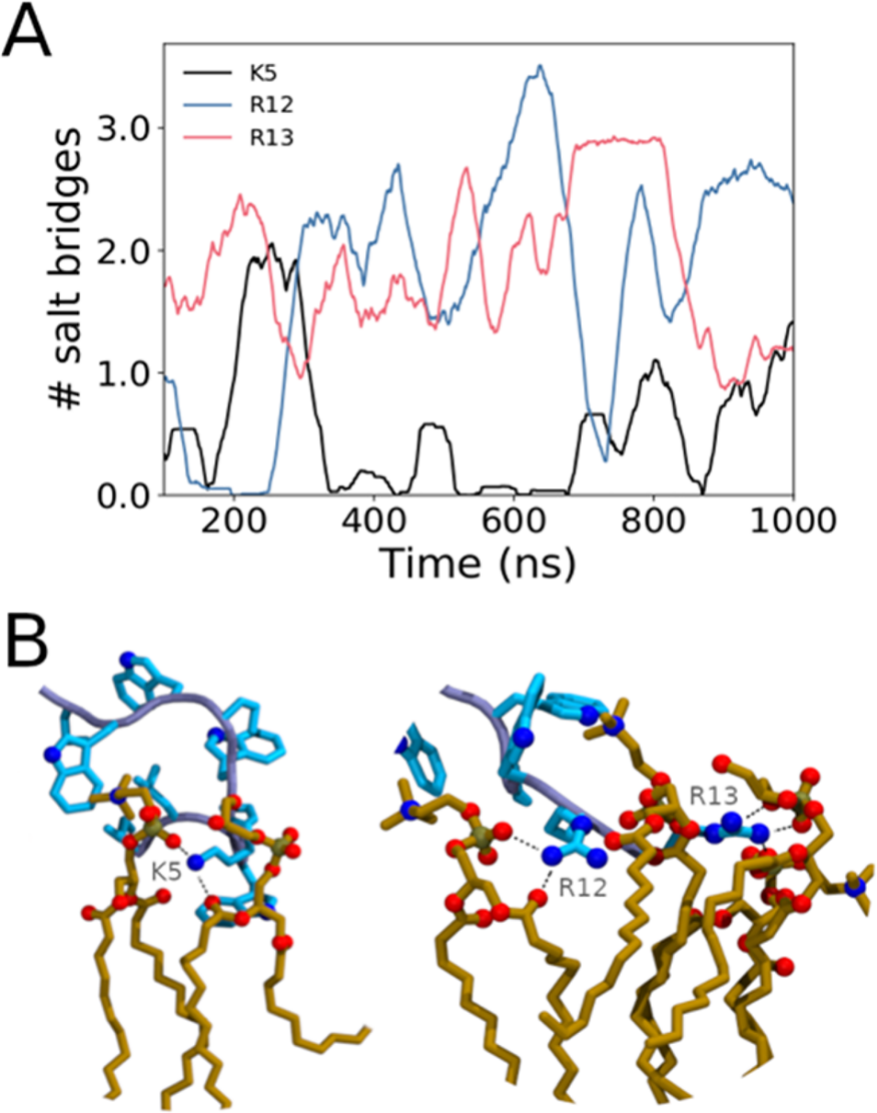
Average number of salt bridges between positively charged peptide side chains and lipid phosphate groups during the 1 μs simulation (A). The time series illustrates the role of Arg12 (blue line), Arg13 (red line), and Lys5 (black line) as peptide anchors to the membrane surface. Panel B showcases structural examples of such interactions. Indolicidin side-chains are shown in cyan. Its backbone trace is shown in light blue, and phospholipid molecules are shown in orange. Relevant nitrogen and oxygen atoms are highlighted as blue and red balls, respectively. Selected salt bridges are shown with black dashed lines.

The main interactions binding the peptide to the lipid membrane are salt bridges among the negatively charged phosphate of the lipids and the positively charged side-chains K5, R12, and R13 of indolicidin (Figure 3). In particular, R12 and R13 at the C-terminus strongly bind to the lipid head groups favoring the peptide penetration into the leaflet with the assistance of the less hydrophilic tryptophan W9 and W11. The average number of salt bridges sampled during our simulations is 4.1 ± 1.7. Salt-bridge interactions with the flipping lipid are kept throughout the initial translocation of the hydrophilic head into the hydrophobic region of the membrane. This is feasible due to the conformational flexibility of the peptide, which remains anchored to the outer polar region of the bilayer. As the hydrophilic head of the lipid penetrates well into the hydrophobic region of the membrane, the peptide–phosphate contact is lost and the peptide relaxes back to its original peripheral position, forming new salt bridges with the heads of the remaining lipids. Detachment of the peptide occurs before the transition state (TS), located at the center of the bilayer, and characterized by the inversion of the lipid tails.

## Transient Poration Accompanies Lipid Flipping

In the catalyzed mechanism, peptide peristaltic fluctuations assist the early penetration of the lipid headgroup through the glycerol layer and allow the gathering of water molecules that follow the flipping lipid inside the membrane. In this case, we can observe a transient water pore connecting the two leaflets in the TS, while the lipid adopts a conformation parallel to the membrane (Figure 2C, top). Formation of stable hydrated pores in free membranes are typically associated with drastic reduction of entropy in the system. The entropy loss is mainly attributed to the reduced possibility of hydrogen bonding for the water molecules in the channel.^21^ However, this is not the case during the flip-flop process catalyzed by AMPs as reported by experimental data where similar results are presented also for peptides that do not deeply penetrate the membrane.^14^ In our simulations, we observe that the penetrating water extensively covers the surface of the peptide, which may alleviate solvent entropy loss, thus facilitating pore formation.

Although several papers discuss membrane deformation upon lipid flipping,^22−24^ in most cases these studies only consider lipid bilayers without added salt. Their main findings are that at the TS, when the flipping lipid is at the center of the membrane, the formation of a pore is necessary, and that the PMFs can be quite steep. When salt is included, the energy barrier increases further (for example, from ∼50 kJ/mol to ∼60 kJ/mol for DMPC at 323 K and with 0.14 M NaCl),^25^ but in this case the authors do not provide a description of the TS structure and instead employ a charge imbalance approach to induce pore formation. This suggests that in the presence of a saline concentration, the TS does not necessarily require a long-lived pore. In fact, in our umbrella simulations for the flipping of a free lipid in the system without AMP we do not observe a stable pore formation, even though the polar lipid head penetrates the bilayer as a solvated moiety (Figure 2C, bottom).

## Entropy-Driven Catalysis of Flip-Flop Rate

Experimentally, the catalytic role of the peptide is associated with an increased activation entropy (ΔΔ*S*^‡^) of the process.^9^ This is evident for several peptides although it may also be accompanied by a decrease in activation enthalpy (ΔΔ*H*^‡^).^9,26^ However, for indolicidin and in particular LL-37, we see a very modest or no reduction in Δ*H*^‡^ respectively. This effect has been elucidated by analysis of the experimental Arrhenius plots obtained from TR-SANS measurements at different temperatures.^9^ This suggests a universal catalytic mechanism played by peptides, despite the differences in their sequences. To rationalize the increase in the activation entropy due to peptide assistance, it is necessary to analyze the structural changes in the whole system during the flip-flop motion. Generally, positive ΔΔ*S*^‡^ can be associated with an increased disordering of the flipping lipid (and neighboring environment) at the TS compared to the optimally organized lipid packing in the membrane leaflet. For indolicidin, DSC showed a significant broadening of the melting transition associated with the heat capacity.^9,17^ This translates also to a small increase in the entropy of fusion, which may be indicative of the role of the peptide.

In our simulations, we find that in the peptide-assisted process, the lipid dissociates from the peptide before reaching the TS, located roughly at the center of the bilayer, likewise to the free flipping case. Hence, the two TSs do not exhibit significant differences in the flipping lipid conformation and mobility. Therefore, the increase in the flipping entropy should be qualitatively attributed to the binding of the peptide to the membrane and to the consequent ordering of the lipids bound to it. The profiles of the potential of mean force indicate that the free energy gain in the peptide-assisted flipping process is associated with the early steps of lipid penetration, corresponding to the initial distortion of the organized packing in the leaflet. Comparing the flipping lipid in the two starting states (free or peptide-bound), binding to the AMP is associated with a loss of mobility, both in terms of translational and conformational contributions. To identify the structural origin of such entropy changes, we characterized the mobility of the flipping lipid in the starting state. The atom positional root-mean-square fluctuations (RMSF) of the lipid show that, when bound, its conformational mobility is only partially lost, with a drop of ∼9% compared to the free lipid case (RMSF/atom = 2.3 and 2.56 Å, respectively). Instead, from the mean square displacement (MSD) of the polar head, we obtain that the lateral diffusion coefficient of the bound lipid is ∼37.5 times smaller than the one of the free lipids (0.4 × 10^–8^ cm^2^ s^–1^ against 1.5 × 10^–7^ cm^2^ s^–1^). This significant drop indicates that the main entropy loss in the starting state is translational. In the uncatalyzed process, unfavorable insertion of the polar head into the lipid bilayer requires a strong deformation of the whole membrane structure, with a significant invagination of the lipid leaflet (Figure 2B). Thus, the peptide assisted early penetration into the leaflet enforces a localization of the flipping lipid at the invagination point with consequent loss of translational entropy, potentially explaining the most significant difference in the observed free energy profile between the two cases.

In conclusion, we have rationalized the mechanism of indolicidin assisted lipid flip-flop and explained the experimental findings regarding the difference in activation barrier between the catalyzed and uncatalyzed systems. The activation free energy reduction is related to both a small reduction in the activation enthalpy, Δ*H*^‡^, and the loss of translational entropy, Δ*S*^‡^, of the flipping lipid interacting with the AMP. This effect is present even at low peptide concentrations. Similar trends were found for other AMPs, and for certain peptides, such as LL-37, the acceleration of the flip-flop dynamics could be solely attributed to an increase in Δ*S*^‡^. This points toward a generic mechanism for interface active peptides that is able to interact with lipids and accelerate the dynamics through a complexing mechanism rather than by the action of an amphiphile which would likely only lower the surface energy. Moreover, this finding elucidates the *transient* rather than *static pore* concept as we show that transport may occur without a transmembrane structured peptide. Our results clearly demonstrate the dynamic nature of poration and show that transient water channels and enhanced lipid flip-flop induced by peptides can be responsible for a minimum mode of action for antimicrobial peptides. These results shed light on the mechanism of antimicrobial peptides and may open the way for the simplified design of new active therapeutics against bacteria strains resistant to conventional antibiotics. Membrane-active therapeutic drugs are relevant beyond bacterial-associated diseases—they are essential antiviral agents, and also used in cancer therapies.^27^ Therefore, it is of crucial importance to be able to tailor the molecular interactions by controlling their activity. Future investigation should involve a detailed understanding of the enthalpic-entropic balance of lipid flipping, as well as its control by point mutations.

## Methods

### System and Simulation Setup

The MD system was set up with a single indolicidin (1G89) positioned on top of a DMPC/DMPG (3:1) membrane in a 150 mM NaCl solution using CHARMM-GUI.^28,29^ The total number of lipids was 512, while the number of TIP3P water molecules was ∼40 000. The box size was approximately 12 × 12 × 12 nm. Simulations were run with a time step of 2 fs at 303.15 K, above the *T*_m_ = 297.35 K reported for this lipid composition,^9,17^ and 1 bar with the CHARMM-36m^2^^30^ force-field in GROMACS 2021.5^31^ using the velocity rescale thermostat^32^ with τ_T_ = 1 ps, and the cell rescale barostat^33^ with τ_P_ = 5 ps. Long range electrostatic interactions were computed with the PME method, with a 1.2 nm real space cutoff and 0.12 Fourier spacing. We used the same cutoff for the Lennard-Jones interactions. Bonds involving hydrogen atoms were constrained with the LINCS algorithm.^34^ Initially, we ran a 1 μs simulation to check the partitioning of indolicidin in our model membrane. For the calculation of the potential of mean force for the lipid flip-flop process, we ran simulations of ∼40 umbrella sampling windows separated by ∼0.08 nm, biasing the position of a lipid P atom with respect to the center of mass of the whole membrane. The starting configurations were obtained via a short 10 ns simulation where the membrane was kept restrained and a DMPC phosphate was pulled with a rate of 0.001 nm ps^–1^. The different windows were run for 100 ns, but only the last 50 ns were used as input for the weighted histogram analysis method (WHAM)^35^ to obtain the free energy profiles. The force constant for the harmonic bias was set to 3000 kJ mol^–1^. Membrane surfaces were calculated with SuAVE.^36,37^

### SAXS Calculations

SAXS spectra were evaluated from the density profiles using SIMtoEXP software,^18^ with an electron density for water of 0.335 e/Å^3^.

For the peptide-bound systems, the density profile was estimated from window 8 of the umbrella sampling simulations, corresponding to the most penetrating conformation of the peptide without an increase in the PMF.

## Data Availability

Simulation data presented in this work are openly available free of charge at the GitHub repository: https://github.com/Cascella-Group-UiO/Publications.
